# Hyperactivity and Inattention in Young Patients Born With an Atrial Septal or Ventricular Septal Defect

**DOI:** 10.3389/fped.2021.786638

**Published:** 2021-12-06

**Authors:** Sara Hirani Lau-Jensen, Benjamin Asschenfeldt, Lars Evald, Vibeke E. Hjortdal

**Affiliations:** ^1^Department of Cardiothoracic Surgery, Rigshospitalet, University of Copenhagen, Copenhagen, Denmark; ^2^Department of Clinical Medicine, Aarhus University, Aalborg, Denmark; ^3^Department of Cardiothoracic and Vascular Surgery, Aarhus University Hospital, Aarhus, Denmark; ^4^Hammel Neurodelvelopmental Center and University Research Clinic, Hammel, Denmark

**Keywords:** neuro-psychiatric disease, young adult, case - control, inattention, inattention deficit hyperactivity disorder, congenital hear defects, morbidity

## Abstract

**Background:** Patients with congenital heart defects have a well-established risk of neuropsychiatric comorbidities. Inattention and hyperactivity are three to four times more frequent in children with complex congenital heart defects. We have previously shown a higher burden of overall attention deficit/hyperactivity disorder (ADHD) symptoms in adults with simple congenital heart defects as well. However, it is unknown whether the higher burden of ADHD symptoms is mainly driven by hyperactivity, inattention, or both.

**Methods:** The participants [simple congenital heart defect = 80 (26.6 years old), controls = 36 (25.3 years old)] and a close relative for each (*n* = 107) responded to the long version of the Conners' Adults ADHD Rating Scales questionnaire. Our primary and secondary outcomes are mean T-scores in the ADHD scores and symptom sub-scores.

**Results:** Patients with simple congenital heart defects reported a higher mean T-score at all three DSM-IV ADHD scores (ADHD—combined: 52.8 vs. 44.9, *p* = 0.007, ADHD—inattention: 55.5 vs. 46.4, *p* = 0.002, and ADHD—hyperactivity: 49.4 vs. 44.0, *p* = 0.03) and in all four ADHD symptom sub-scores (inattention/memory problems: 50.3 vs. 44.2, *p* = 0.001, hyperactivity/restlessness: 49.7 vs. 45.9, *p* = 0.03, impulsivity/emotional lability: 50.0 vs. 41.3, *p* = 0.001, and self-esteem problems: 53.8 vs. 46.3, *p* = 0.003). The results were maintained after the removal of outliers (incongruent responses), albeit the hyperactivity/restlessness ADHD symptom sub-score lost significance. Self- and informant ratings differed significantly on the ADHD—inattention score for the congenital heart defect group, where informants rated the ADHD—inattention scores better than the congenital heart defect patients rated themselves.

**Conclusions:** Patients with a simple congenital heart defect have a higher symptom burden across all ADHD scores and all symptom sub-scores. The higher burden of ADHD is driven by both inattention and hyperactivity symptoms, though the inattention symptoms seem more prominent. Close relatives were less aware of the inattention symptoms than the congenital heart defect patients themselves. Routine screening for ADHD symptoms may be warranted to facilitate adequate help and guidance as these symptoms are easily overlooked.

**Clinical Trial Registration:** www.ClinicalTrials.gov, identifier: NCT03871881.

## Introduction

Congenital heart defects (CHDs) are the most common birth defect found in neonates (5.5–8/1,000 all births) (1, 2). With advancements in diagnostics and treatment, including pre-, peri-, and postoperative care and operation techniques, neonates with CHDs survive until childhood and adulthood (3–6). This alters the research focus from mortality to morbidity. A growing body of literature is found on morbidity in patients with complex CHDs. Less is found on patients with simple CHDs, even though the simple CHD is twice as common as the complex ones (2). Patients with CHDs (both complex and simple) have an increased risk of psychiatric and neurocognitive disabilities throughout life and twice the risk for being unemployed than the background population (7–15).

Attention deficit/hyperactivity disorder (ADHD) symptoms, such as inattention and hyperactivity, are found three to four times more frequent in children with complex CHDs (10). The severity of symptoms is found to be related to a disrupted brain network (16). The Diagnostic and Statistical Manual of Psychiatric Disorders, Edition IV (DSM-IV), published by the American Psychiatric Association, defines three different presentations of ADHD: ADHD—combined, ADHD—inattention, and ADHD—hyperactivity. ADHD—combined is diagnosed when clinically significant symptoms of both inattention and hyperactivity are present. In ADHD—inattention, symptoms of inattention are present without clinically significant symptoms of hyperactivity and *vice versa* in regard to ADHD—hyperactivity. In adults, ADHD has a negative impact on everyday life: ADHD increases the risk of unemployment and thereby lowers the overall income for the affected patient (17–19). Moreover, it decreases the quality of life not only for adults but also for children, adolescents, and their families. The negative effect is not entirely uniform but depends on the character and especially the severity of the ADHD symptoms (20).

We have previously found increased ADHD symptoms in patients with simple CHDs, looking at the overall ADHD score (15). It is unclear if the higher ADHD burden in these patients are driven mostly by hyperactivity or inattention symptoms. As these ADHD presentations differ in how they are perceived by others and differ in problem characteristics (21), it is important to make this distinction to best help the affected patients.

In this study, we aimed to evaluate the self- and informant-reported burden of ADHD symptoms in young adults with simple CHDs and investigate whether the symptoms are driven by sub-scores of inattention, hyperactivity, or both.

## Materials and Methods

The study complies with the Declaration of Helsinki (the World Medical Association, 2013). It is approved by the Danish Central Regional's Committee on Biomedical Research Ethics (chart: 1-10-72-233-17) and the Danish Data Protection Agency (chart: 2012-58-006). It is registered in clinicaltrials.gov (identifier: NCT03871881). All participants provided written informed consent prior to enrollment. The data that support the findings of this study are available from the corresponding author upon reasonable request.

### Study Population

We included patients age >18 years with either an isolated atrial septal defect (ASD) or an isolated ventricular septal defect (VSD). We excluded patients with known syndromes (e.g., Trisomy 21), who had a previous stroke, with recent head trauma, who are pregnant, or who lack sufficient Danish language skills.

All patients had been diagnosed and treated for their CHD by a homogeneous group of anesthetists, cardiologists, and cardiac surgeons at Aarhus University Hospital, a tertiary referral hospital, in the years 1990 to 2000.

The study group was composed of 32 surgically closed VSDs, 34 surgically closed ASDs, 10 percutaneously closed ASDs, and four still open ASDs. The control group is composed of 39 healthy peers matched on age, gender, and educational level (International Standard Classification of Education 2011, ISCED).

Each participant provided a close relative (informant) to participate in the study as well.

Both the participants and their close relatives (informant) were asked to respond to the long version of the Conners' Adult ADHD Rating Scales (CAARS) questionnaire (total number of participants = 218).

Most of the participants in this study (except the participants with percutaneously closed ASDs and with open ASDs) have participated in a previous study by Asschenfeldt et al. (15). Please refer to this study for further detailed information on enrollment/recruitment procedures.

### CAARS Questionnaire

The CAARS questionnaire consists of 66 questions, and it is designed to screen for ADHD symptoms. As an overall parameter, it provides an ADHD index score which is composed of the questions most likely to differentiate a person with ADHD from a person without ADHD. It is further composed of ADHD scores that differentiate between the DSM-IV diagnosis of the three presentations of ADHD:

(1) ADHD—combined, (2) ADHD—inattention, and (3) ADHD—hyperactivity.

It also includes four ADHD symptom sub-scores related to the ADHD diagnosis:

(1) inattention/memory problems, (2) hyperactivity/restlessness, (3) impulsivity/emotional lability, and (4) self-esteem problems.

The CAARS questionnaire is validated, and all ADHD scores and ADHD symptom sub-scores can be standardized to an age- and gender-corrected T-score (mean, 50; SD, 10) based on American normative data. Higher scores indicate a higher presence of symptoms.

An incongruent score for each participant can be calculated. A high incongruent score (>8) is most likely due to an unwilling participant, random answering, or a participant answering to make a specific impression. Exclusion of responses with an incongruent score >8 increases the validity of the answers.

Though the CAARS questionnaire is based on DSM-IV, the definition of ADHD has remained the same from DSM-IV and DSM-V.

Like most other psychiatric questionnaires, CAARS does not, by itself, provide a diagnosis. In this study, we will use the results to provide an estimate of a given burden of symptoms.

### Statistics

The percentage of T-scores above 65 (corresponding to the 93rd percentile), representing a great likelihood for a clinical problem, was calculated for each group.

Data are presented as mean (±SD) or as *n* (%) where appropriate. Comparisons between all three groups were done with ANOVA, and when appropriate, *post-hoc* comparisons between two groups were made with either Welch *T*-test or Student's *T*-test.

Chi-square test was used to analyze a dichotomous outcome.

Our primary outcome was between group difference in the mean T-score of the four DSM-IV ADHD scores: ADHD—index, ADHD—combined presentation, ADHD—inattention presentation, and ADHD—hyperactivity presentation.

*Post-hoc* sub-analyses were done to compare the T-scores of the ADHD symptom sub-scores and to compare the primary outcome between subgroups of the surgically closed ASDs and the percutaneously closed ASDs.

Statistical analysis was performed on a with/without-outlier basis. Analysis of all participants was initially performed regardless of their incongruence score; however, in order to control for potential bias of outliers, patients with an incongruent score above 8 were excluded, and all of the above-mentioned analyses were rerun to test the robustness of the results.

The Benjamini–Hochberg method was used to correct false discovery rates caused by multiple testing.

A *p*-value of 0.05 is considered statistically significant.

#### Sample Size Justification

The sample size was calculated as in the study by Asschenfeldt et al. (15) and estimated on the basis of previously published full-scale IQ data (12). The sample size was determined to be 35 CHD patients to find a difference in IQ (the primary outcome of the previous study) with a power of 80% and a significance level of 0.05.

## Results

### Baseline Characteristics/Demographics

The groups (CHD vs. controls) were comparable on sex/gender, age, body mass index, and ISCED education level (Table 1).

**Table 1:** Demographics.

	**ASD**	**VSD**	**CHD**	**Control**
	**(*n* = 48)**	**(*n* = 32)**	**(*n* = 80)**	**(*n* = 39)**
**Gender (** ***n*** **, %)**				
Male	9 (18.8%)	14 (43.8%)	23 (28.8%)	14 (35.9%)
Female	39 (81.3%)	18 (56.3%)	57 (71.3%)	25 (64.1%)
**Age**				
Mean (SD)	28.7 (6.44)	23.4 (3.34)	26.6 (6.00)	25.3 (4.53)
**Body mass index**				
Mean (SD)	25.7 (5.68)	23.1 (2.96)	24.6 (4.93)	22.9 (3.20)
**Education (** ***n*** **, %)**				
ISCED primary education	2 (4.2%)	1 (3.1%)	3 (3.8%)	0 (0%)
ISCED secondary education	31 (64.6%)	26 (81.3%)	57 (71.3%)	25 (64.1%)
ISCED tertiary education	15 (31.3%)	5 (15.6%)	20 (25.0%)	14 (35.9%)
**Any psychiatric disorder** **^a^ (** ***n*** **, %)**				
Yes	-	-	27 (33.8)	4 (10.3%)
**ADHD, ADD**				
Yes	-	-	7 (8.8%)	0 (0%)
**Informant relationship (** ***n*** **, %)**				
CHD—informant: (*n* = 75), information on *n* = 71	
Control—informant: (*n* = 32), information on all	
Parent	-	-	43 (57.3%)	12 (37.5%)
Spouse, partner	-	-	21 (29.6)	10 (31.3%)
Sibling	-	-	6 (8.5%)	8 (25.0%)
Friend	-	-	0 (0%)	2 (6.3%)
Unanswered	-	-	1 (1.4%)	0 (0%)
**Duration of relation (years)**				
Mean (SD)	-	-	18.9 (9.48)	17.4 (10.5)

*ASD, atrial septum defects; VSD, ventricular septum defect; ISCED, International Standard Classification of Education*.

a *Psychosis, depression, OCD, anxiety, eating disorder, personality disorder, autism spectrum disorders and ADHD/ADD*.

The participants gave a self-reported status of prior psychiatric diagnosis (Table 1). More of the participants in the CHD group had a prior psychiatric diagnosis than the participants in the control group.

As there were no differences in the mean T-scores between the VSD and the ASD group in any of the ADHD scores or symptom sub-scores, we analyzed these two groups combined as a CHD group (Table 2).

**Table 2:** Mean and SD T-scores.

**DMS-IV**	**ASD**	**VSD**	**CHD**	**Control**	**ASD vs. VSD**^a^****	**CHD vs. control**^a^****	**Hedges' g**
**ADHD index score**							
Mean (SD)	54.0 (12.3)	53.6 (12.6)	53.8 (12.4)	45.1 (9.2)	0.9	0.001^*^	0.76
**ADHD—combined**							
Mean (SD)	51.6 (13.0)	54.5 (14.4)	52.8 (13.6)	44.9 (10.5)	0.3	0.007^*^	0.62
**ADHD—inattention**							
Mean (SD)	54.1 (12.7)	57.7 (13.8)	55.5 (13.2)	46.4 (11.4)	0.2	0.002^*^	0.72
**ADHD—Hyperactivity**							
Mean (SD)	48.8 (12.7)	50.3 (13.8)	49.4 (13.1)	44.0 (8.1)	0.6	0.03^*^	0.46
**Symptoms**							
**Inattention/memory problems**							
Mean (SD)	50.5 (9.0)	50.3 (11.7)	50.3 (10.1)	44.2 (7.2)	0.8	0.001^*^	0.66
**Hyperactivity/restlessness**							
Mean (SD)	49.0 (9.2)	50.7 (10.0)	49.7 (9.5)	45.9 (7.3)	0.4	0.03^*^	0.43
**Impulsivity/emotional lability**							
Mean (SD)	50.3 (12.2)	49.6 (14.5)	50.0 (13.1)	41.3 (9.0)	0.8	0.001^*^	0.73
**Self-esteem problems**							
Mean (SD)	55.2 (12.7)	51.7 (11.1)	53.8 (12.1)	46.3 (10.5)	0.2	0.003^*^	0.65

*ASD, atrial septum defect; VSD, ventricular septum defect; CHD, congenital heart defect*.

a *T-test*.

* *p < 0.05 (after multiple analysis correction; Benjamini–Hochberg correction)*.

### T-Score >65

**Figure 1:**
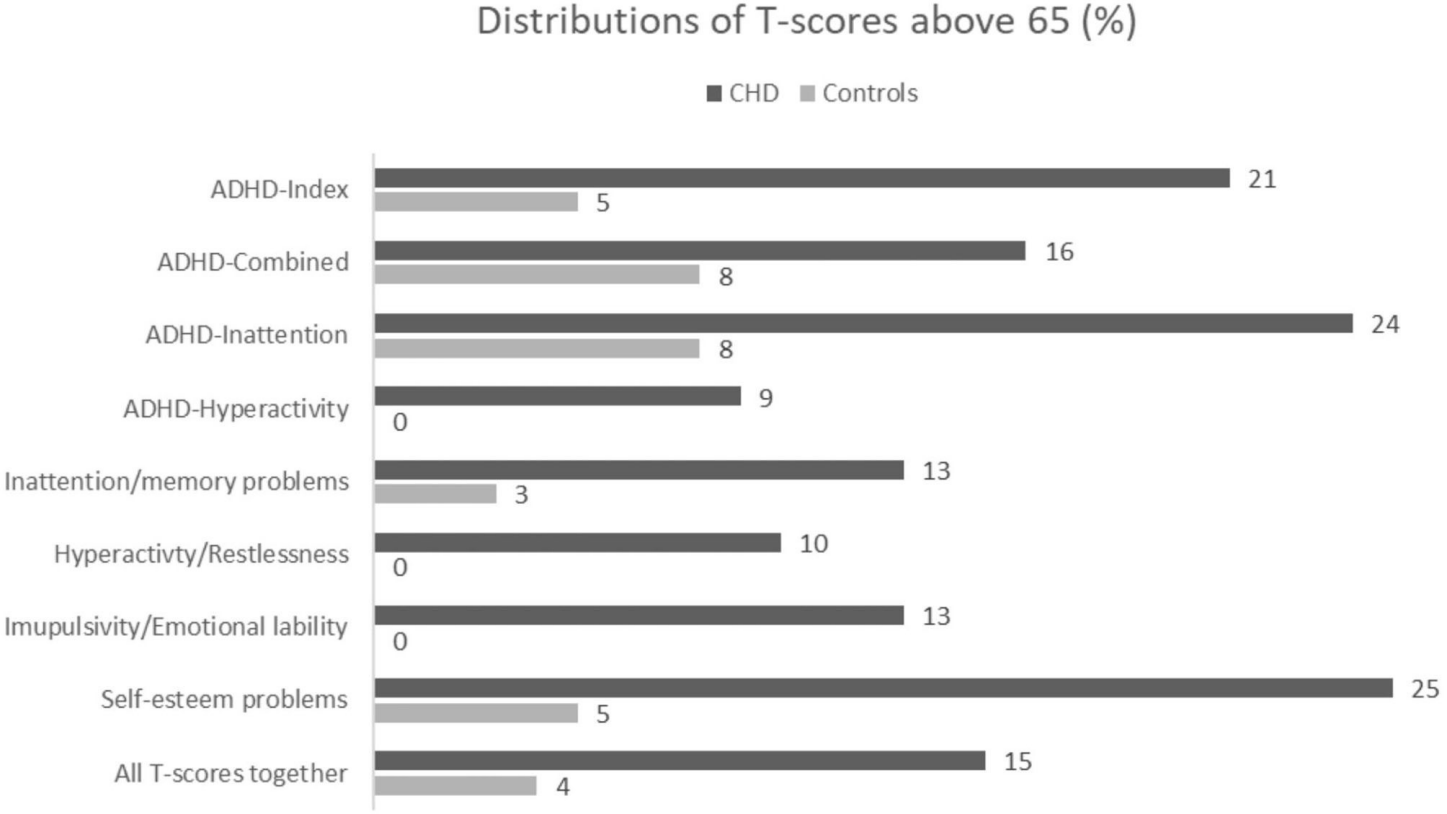
Distribution in % of T-scores above 65 between group for all ADHD-Index score,ADHD DSM-IV scores, all symptoms sub-scores and all scores together.

In the CHD group, 15% of all T-scores were above 65 compared to 4% of the T-scores in the control group [RR = 4.61 (2.52, 8.5), *p* < 0.001] (Figure 1). If a participant has one or more T-scores above 65, the likelihood of a diagnosis of ADHD is higher. We found that a total of 31 participants (38.8%) in the CHD group had more than one T-score above 65 (Table 3).

**Table 3:** Participants at risk for an ADHD diagnosis between the CHD group and the control group.

	**CHD (*N* = 80)**	**Control (*N* = 39)**
**Count of T-scores above 65**		
0	49 (61.3%)	35 (89.7%)
1–8	31 (38.8%)	4 (10.3%)

### DSM-IV ADHD Scores

We performed a *post-hoc* analysis of the different ADHD scores between the CHD and the control groups to characterize this difference in continuous T-scores.

The CHD group reported significantly higher means across all ADHD scores than the control group (Table 2).

**Figure 2:**
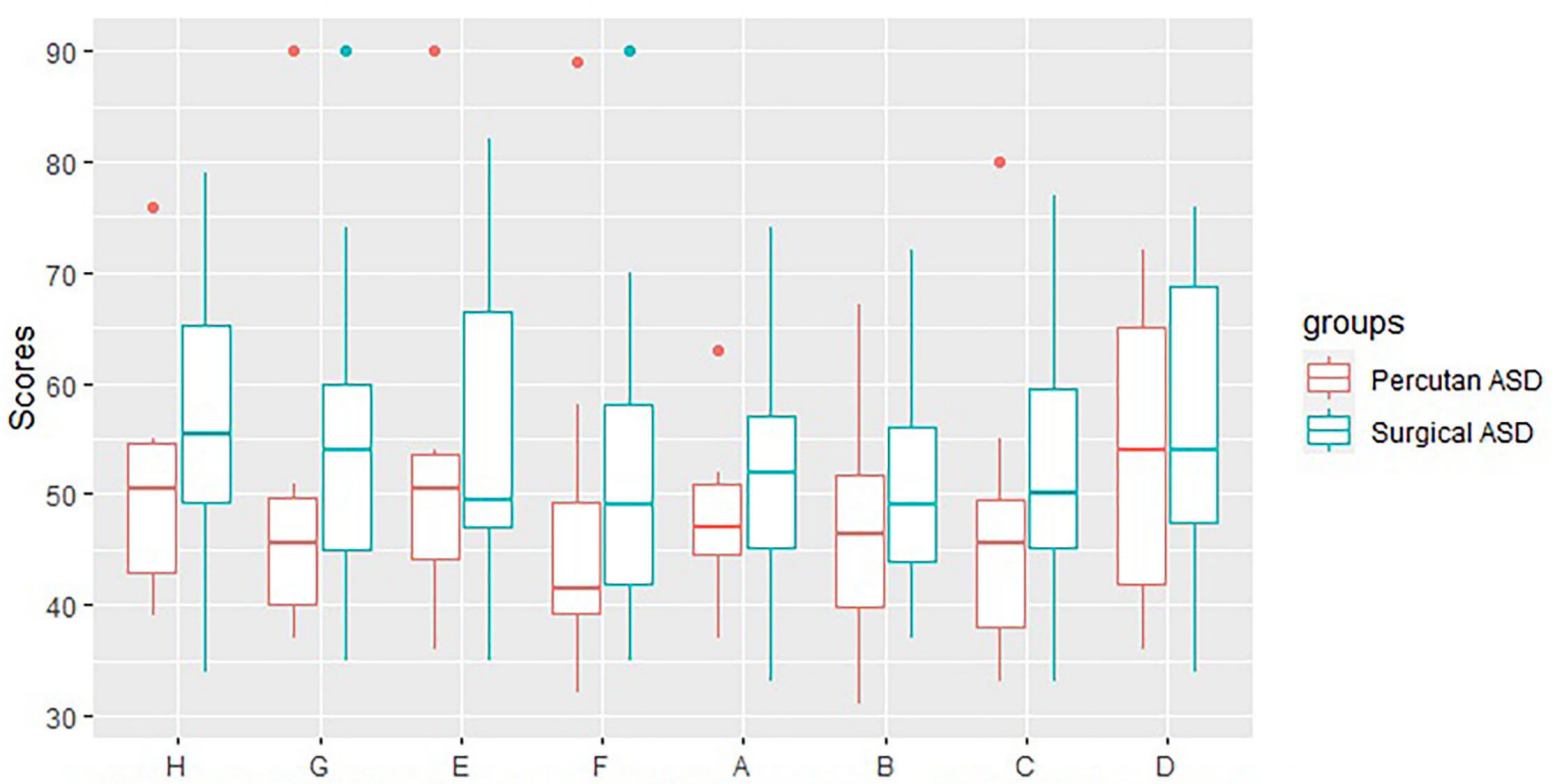
CAARS scores for Perctuaneus ASD and Surgical ASD. H: ADHD-Index, G: ADHD-Combined, E: ADHD-Inattention, F: ADHD-Hyperactivity, A: Innatention/Memory problems, B: Hyperactivity/Restlessness, C: Impulsivity/Emotional lability, D: Self-esteem problems.

We found no difference in the ADHD scores between the subgroup of ASD patients treated with percutaneous ASD closure (*N* = 10) compared to ASD patients treated with open surgery (*N* = 34) (ADHD index score: 50.7 vs. 55.8, *p* = 0.3, ADHD—combined: 48.8 vs. 53.4, *p* = 0.3, ADHD—inattention: 52.1 vs. 55.5, *p* = 0.5, and ADHD—hyperactivity: 47.0 vs. 50.3, *p* = 0.5) (Figure 2).

### ADHD Symptoms Sub-scores

The CHD group reported a higher mean T-score in all of the four ADHD symptom sub-scores than the control group (Table 2).

Hedges' *g* effect size was calculated, and a medium effect size between groups on all ADHD scores and three out of four ADHD symptom sub-scores (inattention/memory problems, impulsivity/emotional lability, and self-esteem problems) were found. Only a small effect size was found on hyperactivity/restlessness symptoms (Table 2).

### Self-Report vs. Informant Report

Compared to the informant report, the CHD group reported themselves as having more problems at the ADHD—inattention score, but not at the ADHD—hyperactivity score and ADHD—combined score (Table 4). No differences were found between self- and informant-reported ADHD symptom sub-scores. There were no differences between self- and informant-reported scores in the control group (Table 4).

**Table 4:** Congenital heart defect informant vs. congenital heart defect self-evaluation (mean and SD of T-scores).

**CHD**				**Control**		
**DMS-IV**	**Self**	**Informant**	**Self vs. informant**^a^****	**Self**	**Informant**	**Self vs. Informant**^a^****
**ADHD index score**						
Mean (SD)	53.8 (12.3)	49.7 (11.4)	0.16	45.1 (9.2)	45.2 (8.1)	0.94
**ADHD—combined score**						
Mean (SD)	52.8 (13.6)	46.0 (9.1)	0.08	44.9 (20.5)	43.1 (7)	0.37
**ADHD—inattention score**						
Mean (SD)	55.5 (13.2)	46.9 (8.5)	0.003^*^	46.6 (11.4)	44.3 (7.3)	0.3
**ADHD—hyperactivity score**						
Mean (SD)	49.4 (13.1)	45.1 (9.9)	0.08	44.0 (8.1)	42.7 (6)	0.45
**Symptoms**						
**Inattention/memory problems**						
Mean (SD)	50.3 (10.1)	48.6 (11.0)	0.35	44.0 (7.5)	45.3 (7.6)	0.48
**Hyperactivity/restlessness**						
Mean (SD)	49.7 (9.5)	47.1 (9.4)	0.35	45.9 (7.3)	45.2 (7.4)	0.69
**Impulsivity/emotional lability**						
Mean (SD)	50.0 (13.1)	48.0 (9.6)	0.35	41.3 (9.0)	43.2 (5.8)	0.31
**Self-esteem problems**						
Mean (SD)	52.3 (12.0)	50.9 (10.9)	0.35	46.3 (10.5)	48.4 (9.6)	0.4

a *T-test*.

* *p < 0.05 after multiple analysis correction (Benjamini–Hochberg correction)*.

### Excluding Incongruent Responses

Based on the CAARS incongruent subscore, a total of 14 participants were excluded (two percutaneously closed ASDs, five surgically closed ASDs, four surgically closed VSDs, and three controls), leaving a total number of 36 controls, 28 VSDs, and 41 ASDs (29 surgically closed ASDs, eight percutaneously closed ASDs, and four non-operated ASDs).

We again found no difference in the DSM-IV ADHD scores between the subgroup of ASD patients treated with open surgery compared to ASD patients treated with percutaneous ASD closure (ADHD Index score: 53.9 vs. 50.5, *p* = 0.50, ADHD—combined: 51.4 vs. 48.4, *p* = 0.7, ADHD—inattention: 53.1 vs. 53.3, *p* = 1.0, and ADHD—hyperactivity: 49.5 vs. 45.1, *p* = 0.5).

The CHD group reported a significantly higher means across all DSM-IV ADHD scores than the control group (Table 5). The CHD group likewise reported a higher mean T-score in three of the four ADHD symptom sub-scores than the control group, though we found no difference in mean T-scores in the hyperactivity sub-score (Table 5).

**Table 5:** Mean and SD between groups of T-scores (when participants with incongruent answers are excluded).

**Without incongruent answers**	**ASD**	**VSD**	**CHD**	**Control**	**ASD vs. VSD**^a^****	**CHD vs. control**^a^****	**Hedges' g**
**DSM-IV ADHD scores**							
**ADHD index score**							
Mean (SD)	52.5 (11.9)	51.6 (12.0)	52.2 (11.9)	44.9 (9.4)	0.74	0.01^*^	0.66
**ADHD—combined score**							
Mean (SD)	50.0 (13.0)	52.9 (13.6)	51.2 (13.2)	44.3 (10.5)	0.38	0.02^*^	0.56
**ADHD—inattention score**							
Mean (SD)	52.5 (12.3)	56.1 (12.8)	54.0 (12.5)	45.9 (11.6)	0.25	0.01^*^	0.66
**ADHD—hyperactivity score**							
Mean (SD)	47.7 (13.2)	48.8 (12.5)	48.1 (12.9)	43.5 (8.0)	0.74	0.05^*^	0.4
**Symptoms sub-scores**							
**Inattention/memory problems**							
Mean (SD)	49.2 (8.4)	48.0 (10.9)	48.7 (9.4)	43.8 (7.7)	0.6	0.02^*^	0.55
**Hyperactivity/restlessness**							
Mean (SD)	47.8 (8.9)	49.2 (9.4)	48.4 (9.1)	45.4 (7.1)	0.53	0.12	0.35
**Impulsivity/emotional lability**							
Mean (SD)	49.7 (12.5)	47.8 (14.4)	49.0 (13.3)	41.1 (9.2)	0.56	0.01^*^	0.65
**Self-esteem problems**							
Mean (SD)	53.7 (12.5)	50.4 (11.0)	52.3 (12.0)	46.2 (10.8)	0.26	0.03^*^	0.53

*Number of participants with incongruent answers: ASD = 7, VSD = 4, and controls = 3*.

*ASD, atrial septum defect; VSD ventricular septum defect; CHD, congenital heart defect*.

a *T-test*.

* *p < 0.05 after multiple analysis correction*.

The 14 participants excluded in the above-mentioned analysis had a higher mean T-score on all scores and sub-scores both for the CHD group and for the control group [ADHD index score: 64.2 (SD 10.7) and 46.7 (SD 6.1), ADHD-combined score: 62.5 (SD 11.8) and 53.3 (SD 7.0), ADHD—inattention: 65.3 (SD 13.8) and 54.7 (SD 5.5), and ADHD—hyperactivity: 57.0 (SD 8.1) and 50.3 (8.1), inattention/memory problems: 60.6 (SD 8.1) and 47.3 (SD 3.1), impulsivity/emotional lability: 56.7 (SD 10.1) and 44.7 (SD 7.8), self-esteem problems: 62.9 (SD 9.5), and 47.7 (SD 8.4), hyperactivity/restlessness: 57.6 (SD 8.6) and 52.0 (SD 8.0)]. Moreover, 34% of T-scores in the excluded CHD group were above 65 vs. 0% in the excluded control group.

#### Self-Report vs. Informant Report Without Participants With Incongruent Answers

The CHD group reported themselves as having more problems at the ADHD—combined score and at the ADHD—inattention score, but not at the ADHD—hyperactivity score, compared to the informant report. There were no differences between self- and informant-reported scores in the control group (Table 6).

**Table 6:** Congenital heart defect informant vs. congenital heart defect self-evaluation.

**Without incongruent answers**				**Controls**		
**CHD**						
**DMS-IV ADHD scores**	**Self**	**Informant**	**Self vs. informant** **^a^**	**Self**	**Informant**	**Self vs. Informant** **^a^**
**ADHD index score**						
Mean (SD)	52.2 (11.9)	48.1 (10.4)	0.3	44.9 (9.4)	45.7 (8.1)	0.73
**ADHD—combined score**						
Mean (SD)	51.2 (13.2)	42.2 (7.5)	0.03^*^	44.3 (10.5)	43.4 (7.1)	0.66
**ADHD—inattention score**						
Mean (SD)	54.0 (12.5)	45.6 (7.7)	0.002^*^	45.9 (11.6)	44.4 (7.5)	0.52
**ADHD—hyperactivity score**						
Mean (SD)	48.1 (12.9)	43.1 (8.4)	0.06	43.5 (8.0)	43 (6.1)	0.79
**Symptom sub-scores**						
**Inattention/memory problems**						
Mean (SD)	48.7 (9.4)	46.8 (9.3)	0.46	43.8 (7.7)	45.7 (7.7)	0.36
**Hyperactivity/restlessness**						
Mean (SD)	48.4 (9.1)	45.2 (7.7)	0.06	45.4 (7.1)	45.5 (7.6)	0.95
**Impulsivity/emotional lability**						
Mean (SD)	49.0 (13.3)	46.5 (8.6)	0.3	41.1 (9.2)	43.5 (5.7)	0.2
**Self-esteem problems**						
Mean (SD)	52.3 (12.0)	50.9 (10.9)	0.46	46.2 (10.8)	48.8 (9.7)	0.31

*Mean and SD of T-scores (when participants with incongruent answers are excluded)*.

a *T-test*.

* *p < 0.05 after multiple analysis correction*.

## Discussion

The aim of this study was to investigate the distribution in ADHD symptoms in young adults with simple CHD and investigate whether such a burden is mostly driven by inattention, hyperactivity, or both.

We found that the CHD group had a higher burden of ADHD symptoms in general (all of ADHD—combined, ADHD—inattention, and ADHD—hyperactivity) and all related symptoms (inattention/memory problems, impulsivity/emotional lability, hyperactivity/restlessness, and self-esteem problems) compared to healthy controls.

Nevertheless, this burden seems somewhat more dominated by inattention and less by hyperactivity symptoms, as the hyperactivity sub-scores reveal smaller effect sizes and did not reach significance after removing the incongruent responses. Typically, inattention symptoms continue into adulthood, whereas hyperactivity seems to burn out with age. This could be why we only find a small effect size in this group of young adults. In accordance to our findings, a higher burden of inattention symptoms, but not hyperactivity, has previously been documented in a combined group of complex (TGA) and simple (VSD) CHD children (age 10–17) when compared to a matched control group (22). We found both a higher burden of inattention and hyperactivity while not excluding the incongruent responses. These findings indicate that some participants in our CHD group have more hyperactivity symptoms that linger into adulthood despite the general adult ADHD presentation. When analyzing the participants with incongruent responses, we see that these participants have higher mean T-scores—especially in the CHD group. Two (11%) of the participants in the CHD group who had incongruent answers had a prior diagnosis of ADHD/ADD. We found that 34% of all T-scores in this group were above the 93rd percentile (corresponding to a great likelihood for clinical problems) compared to 15% for the CHD group as a whole. This suggests that the incongruent responses may be part of their symptomatology (or a desire to make a specific impression) and not due to unwillingness to answer the questions. More of the participants in the CHD group had a prior psychiatric diagnosis of any kind (33.8 vs. 10.3%) and more of them had a prior specific diagnosis of ADHD/ADD (8.8 vs. 0%) than the participants in the control group. The higher frequency of preexisting psychiatric diagnoses and our findings of previously undiscovered symptoms confirm that the psychiatric well-being of young adults with CHD is challenged.

The informants reported the CHD patients better (i.e., lower scores) than the CHD patients reported themselves at the scores for ADHD—inattention. There are different possibilities as to why we find this discrepancy between participants and informants. It could be that the participants over-report their problems because of symptoms of depression or general low self-worth. The discrepancy could also be due to informants being defensive in their responses (“things are better than I expected”), or it could be that the informants are not aware of the struggling at school or work because inattention problems are more “silent” than hyperactivity. Since the discrepancy is only seen with regards to ADHD—inattention, we think the latter to be most likely in this study. This corresponded well with another study that found that parents are less aware of the social difficulties experienced by children with ADHD—inattention (21). ADHD—inattention is associated with difficulties in academic achievements compared to ADHD—hyperactivity which is more associated with behavioral impairments that may be more evident at home (23). This correlated well with our findings. The problems of this patient group with inattention may not be apparent for other people—even people close to the patient. Awareness of potential inattention is the first step in managing these difficulties in a child or an adolescent. In this aspect, the treating physician has a special and important role in making the parent or other close relatives aware of these difficulties and their effect on academic achievements.

ADHD—combined and ADHD—inattention are associated with problems in social performance. ADHD—inattention is associated with social performance problems linked to more passive behaviors. ADHD—combined is associated with more aggressive behavior problems, which, in turn, are more visible to the outside world (21). Inattention symptoms are associated with negative performance on stimuli inhibition, vigilance, and processing speed (more than hyperactivity symptoms) (24, 25). These problems could explain the issues with social cognition found in patients with simple CHD (15). Most studies trying to describe the difference in cognitive and social functions between subtypes have focused on ADHD—inattention and ADHD—combined. Less have been made on ADHD—hyperactivity, and the results are conflicting. One study found that children with ADHD—hyperactivity have been found to have the same level of executive functioning and response inhibition as those with ADHD—inattention (26). Another study found that ADHD—hyperactivity had the best cognitive functioning and the best self-rated self-esteem of the subtypes (27).

Both patients with complex and simple CHDs have neurocognitive and psychiatric comorbidities compared to the general population (7–15). Our findings are in line with these previous studies. Different pathophysiological pathways have been suggested to explain these comorbidities, including brain development, brain hypoxia, brain vulnerabilities, surgical complications, and genetics.

Different pre-, peri-, and post-surgical complications are known risk factors for early and late neuropsychiatric comorbidities in patients with complex CHD (28, 29). Research has progressed to also include patients with simple CHD that only requires minor operations, and even some without any operations (7) and surgical complications cannot explain the neuropsychiatric complications detected in this patient group. In our study, we found no difference in ADHD scores between patients with surgically corrected ASDs and percutaneously corrected ASDs. However, our subgroups are small (ASD surgically corrected: *N* = 34 and ASD percutaneously corrected: *N* = 10). These findings are in line with the above-mentioned notion that other factors than surgical complications may play a bigger role in the development of neurocognitive and psychiatric morbidities in simple CHDs.

In the third trimester of pregnancy, the development of the brain is accentuated, and MRI scans *in utero* have shown delayed brain maturation, decreased white and gray volumes, immature cortical gyrification, and affected brain metabolism (30–32). Related findings have also been found in newborns pre-operatively (33–35). Moreover, in a recently published study from our group, we found abnormal sulcal folding patterns in adults with simple CHDs (36). This supports a notion of a disruption in early brain development in these patients. The general perception is that some of the CHDs result in desaturated blood being delivered to the brain despite cerebral compensatory mechanisms (37). *De novo* damage to specific genes that are involved in both cardiac and neuro-development and function has been found in patients with complex CHD (38). These findings add to the pathophysiological pathways explaining the brain vulnerabilities and neuropsychological difficulties found in the patient group. Genetics could also very well be a contributor to the explanation of the poorer neuropsychological outcomes found in simple CHDs (39). Further research to investigate the possible pathophysiology behind the poorer neuropsychological long-term outcomes in simple CHDs is still needed.

A proper diagnosis of the three different ADHD presentations requires a psychiatric evaluation, and prevalence based on symptom criterions (such as CAARS) must be interpreted with caution since symptom criterions tend to overestimate (23). If a participant has one or more T-scores above 65, the likelihood of a diagnosis of ADHD is higher. We found that a total of 31 participants (38.8%) in the CHD group had more than one T-score above 65 despite only 7 (8.8%) were diagnosed with a ADHD/ADD diagnosis already. An explanation could be that the diagnosis of ADHD/ADD requires a psychiatric evaluation, and especially inattention problems are more difficult to detect. Therefore, some of the participants may not have been correctly recognized and referred to a psychiatrist.

We did not include information on socioeconomic status. We deal with young adults who have not yet established their own socioeconomic status, but they are most likely not adequately defined by the status of their parents. Therefore, we chose to match the control group to the CHD group by the educational level of the patients in the belief that the educational level would be the most accurate reflection of a lifetime socioeconomic status. Moreover, in a large meta-analysis, no correlation was found between socioeconomic status and the prevalence of ADHD (23), but the research is not consistent (40–43).

The observations made by the participant informants are surrogates for a proper psychiatric evaluation that would lead to a definitive ADHD diagnosis. In this light, we avoid extrapolating the results beyond statements about the difference in burden of ADHD and of related symptoms. As the participants in the CHD group are relatively well functioning, the burden of ADHD and symptoms and the quality of this burden were, in fact, what we wanted to explore.

A notable strength of this study is the control group of healthy peers matched on gender, age, and educational level instead of only the normative data provided in the CAARS manual. The CHD group is exclusively simple CHD (ASD and VSD) and not a mixture as most previous studies are. Another strength is that we had evaluations from both the participants themselves and a close relative, adding an extra layer to the understanding. Despite this being a relatively small study, a clinically significant difference was demonstrated.

Our control group had fewer high T-scores (above the 93rd percentile) than expected (4 vs. 7%). In general, the mean T-score for our control group was lower than the normative data provided by the CAARS manual (mean, 50; SD, 10). This is an expected finding as this is the case in many European and especially Scandinavian normative datasets (44–47). This emphasizes the importance of using a comparable control group. The Danish normative data for the CAARS questionnaire has not yet been established. To compensate for this somewhat expected difference in means between our Danish patient group, we used an age- and gender-matched control group as a comparison.

## Conclusion

Young adults with simple CHD have a higher burden of ADHD compared with healthy peers. The difference is driven both by introvert symptoms, like inattention, and also extrovert symptoms (atypically for the age group), like hyperactivity, though the inattention symptoms seem more prominent, and these are more likely to be overlooked by close relatives. Awareness of potential inattention is the first step to managing these difficulties in a child or an adolescent. In this aspect, the treating physician has a special and important role in making the parent or other close relatives aware of these difficulties and their effect on academic achievements.

This study emphasizes the long-term neurodevelopmental struggles of patients with simple CHDs and underlines the importance of raising awareness of these hitherto unrecognized life-changing burdens—struggles that we, as healthcare providers (be it nurses or doctors), need to discuss with our patients and their families.

## Data Availability Statement

The raw data supporting the conclusions of this article will be made available by the authors, without undue reservation.

## Ethics Statement

The studies involving human participants were reviewed and approved by Danish Central Regional's Committee on Biomedical Research Ethics (chart: 1-10-72-233-17). The patients/participants provided their written informed consent to participate in this study.

## Author Contributions

SL-J has contributed with data management, data analyzes and all manuscript writing. BA has conceptualised and designed the study and contributed with funding applications, data collection, input regarding data analyzes, supervision regarding interpretation of results and supervision with manuscript writing. LE and VH have conceptualised and designed the study, they have contributed with general and detailed supervision in all aspects of the data management and manuscript writing.

## Funding

SL-J was funded by The Danish Heart Foundation. The funding payed the salary for the SL-J, but the funder was not included in any aspect of data management, analysis, interpretation or manuscript writing.

## Conflict of Interest

The authors declare that the research was conducted in the absence of any commercial or financial relationships that could be construed as a potential conflict of interest.

## Publisher's Note

All claims expressed in this article are solely those of the authors and do not necessarily represent those of their affiliated organizations, or those of the publisher, the editors and the reviewers. Any product that may be evaluated in this article, or claim that may be made by its manufacturer, is not guaranteed or endorsed by the publisher.

## References

[1] Khoshnood B Loane M Garne E Addor M-C Arriola L Bakker M. Recent decrease in the prevalence of congenital heart defects in Europe. J Pediatr. (2013) 162:108–13.e2. 10.1016/j.jpeds.2012.06.035 22835879

[2] Dolk H Loane M Garne E. Congenital heart defects in Europe: Prevalence and perinatal mortality, 2000 to 2005. Circulation. (2011) 123:841–9. 10.1161/CIRCULATIONAHA.110.958405 21321151

[3] Botto LD Correa A Erickson JD. Racial and temporal variations in the prevalence of heart defects. Pediatrics. (2001) 107:E32. 10.1542/peds.107.3.e32 11230613

[4] Warnes CA Liberthson R Danielson GK Dore A Harris L Hoffman JI. Task force 1: the changing profile of congenital heart disease in adult life. J Am Coll Cardiol. (2001) 37:1170–5. 10.1016/S0735-1097(01)01272-4 11300418

[5] Marelli AJ Mackie AS Ionescu-Ittu R Rahme E Pilote L. Congenital heart disease in the general population: changing prevalence and age distribution. Circulation. (2007) 115:163–72. 10.1161/CIRCULATIONAHA.106.627224 17210844

[6] Marelli AJ Ionescu-Ittu R Mackie AS Guo L Dendukuri N Kaouache M. Lifetime prevalence of congenital heart disease in the general population from 2000 to 2010. Circulation. (2014) 130:749–56. 10.1161/CIRCULATIONAHA.113.008396 24944314

[7] Olsen M Sørensen HT Hjortdal VE Christensen TD Pedersen L. Congenital heart defects and developmental and other psychiatric disorders: A Danish nationwide cohort study. Circulation. (2011) 124:1706–12. 10.1161/CIRCULATIONAHA.110.002832 21947292

[8] Nyboe C Udholm S Larsen SH Rask C Redington A Hjortdal V. Risk of lifetime psychiatric morbidity in adults with atrial septal defect (from a nation-wide cohort). Am J Cardiol. (2020) 128:1–6. 10.1016/j.amjcard.2020.04.047 32650900

[9] Wernovsky G. Current insights regarding neurological and developmental abnormalities in children and young adults with complex congenital cardiac disease. Cardiol Young. (2006) 16 Suppl 1:92–104. 10.1017/S1047951105002398 16401370

[10] Shillingford AJ Glanzman MM Ittenbach RF Clancy RR Gaynor JW Wernovsky G. Inattention, hyperactivity, and school performance in a population of school-age children with complex congenital heart disease. Pediatrics. (2008) 121:e759–767. 10.1542/peds.2007-1066 18381503

[11] Sarrechia I Miatton M François K Gewillig M Meyns B Vingerhoets G. Neurodevelopmental outcome after surgery for acyanotic congenital heart disease. Res Dev Disabil. (2015) 45–6:58–68. 10.1016/j.ridd.2015.07.004 26210851

[12] Schaefer C von Rhein M Knirsch W Huber R Natalucci G Caflisch J. Neurodevelopmental outcome, psychological adjustment, and quality of life in adolescents with congenital heart disease. Dev Med Child Neurol. (2013) 55:1143–9. 10.1111/dmcn.12242 23937239

[13] DeMaso DR Calderon J Taylor GA Holland JE Stopp C White MT. Psychiatric disorders in adolescents with single ventricle congenital heart disease. Pediatrics. (2017) 139:e20162241. 10.1542/peds.2016-2241 28148729 PMC5330395

[14] Nyboe C Fonager K Larsen ML Andreasen JJ Lundbye-Christensen S Hjortdal V. Effect of atrial septal defect in adults on work participation (from a nation wide register-based follow-up study regarding work participation and use of permanent social security benefits). Am J Cardiol. (2019) 124:1775–9. 10.1016/j.amjcard.2019.08.041 31590912

[15] Asschenfeldt B Evald L Heiberg J Salvig C Østergaard L Dalby RB. Neuropsychological status and structural brain imaging in adults with simple congenital heart defects closed in childhood. J Am Heart Assoc. (2020) 9:e015843. 10.1161/JAHA.120.015843 32427039 PMC7428999

[16] Schmithorst VJ Panigrahy A Gaynor JW Watson CG Lee V Bellinger DC. Organizational topology of brain and its relationship to ADHD in adolescents with d-transposition of the great arteries. Brain Behav. (2016) 6:e00504. 10.1002/brb3.504 27547505 PMC4980474

[17] Biederman J Faraone S V. The effects of attention-deficit/hyperactivity disorder on employment and household income. MedGenMed Medscape Gen Med. (2006) 8:12. 17406154 PMC1781280

[18] Fletcher JM. The effects of childhood ADHD on adult labor market outcomes. Health Econ. (2014) 23:159–81. 10.1002/hec.2907 23427026 PMC6714576

[19] Daley D Jacobsen RH Lange AM Sørensen A Walldorf J. The economic burden of adult 35 attention deficit hyperactivity disorder: a sibling comparison cost analysis. Eur Psychiatry. (2019) 61:41–8. 10.1016/j.eurpsy.2019.06.011 31288209

[20] Klassen AF Miller A Fine S. Health-related quality of life in children and adolescents who have a diagnosis of attention-deficit/hyperactivity disorder. Pediatrics. (2004) 114:e541–7. 10.1542/peds.2004-0844 15520087

[21] Maedgen JW Carlson CL. Social functioning and emotional regulation in the attention deficit hyperactivity disorder subtypes. J Clin Child Adolesc Psychol. (2000) 29:30–42. 10.1207/S15374424jccp2901_4 10693030

[22] Holst LM Kronborg JB Jepsen JRM Christensen JO Vejlstrup NG Juul K. Attention-deficit/hyperactivity disorder symptoms in children with surgically corrected ventricular septal defect, transposition of the great arteries, and tetralogy of fallot. Cardiol Young. (2020) 30:180–7. 10.1017/S1047951119003184 31928549

[23] Willcutt EG. The prevalence of DSM-IV attention-deficit/hyperactivity disorder: a meta-analytic review. Vol 9, Neurotherapeutics Neurotherapeutics. (2012) 29:529–40. 10.1007/s13311-012-0135-8 22976615 PMC3441936

[24] Chhabildas N Pennington BF Willcutt EG. A comparison of the neuropsychological profiles of the DSM-IV subtypes of ADHD. J Abnorm Child Psychol. (2001) 29:529–40. 10.1023/a:1012281226028 11761286

[25] Solanto MV Gilbert SN Raj A Zhu J Pope-Boyd S Stepak B. Neurocognitive functioning in AD/HD, predominantly inattentive and combined subtypes. J Abnorm Child Psychol. (2007) 35:729–44. 10.1007/s10802-007-9123-6 17629724 PMC2265203

[26] Ahmadi N Mohammadi MR Araghi SM Zarafshan H. Neurocognitive Profile of children with attention deficit hyperactivity disorders (ADHD): a comparison between subtypes. Iran J Psychiatry. (2014) 9:197–202. 25792987 PMC4361821

[27] Molavi P Nadermohammadi M Ghojehbeiglou HS Vicario CM Nitsche MA Salehinejad MA. Subtype-specific cognitive correlates and association with self-esteem: a quantitative difference. BMC Psychiatry. (2020) 20:502. 10.1186/s12888-020-02887-4 33046041 PMC7549239

[28] Sananes R Manlhiot C Kelly E Hornberger LK Williams WG Macgregor D. Neurodevelopmental outcomes after open heart operations before 3 months of age. ATS. (2012) 93:1577–83. 10.1016/j.athoracsur.2012.02.011 22541188

[29] Sugimoto A Ota N Ibuki K Miyakoshi C Murata M Tosaka Y. Risk factors for adverse neurocognitive outcomes in school-aged patients after the fontan operation. Eur J Cardiothorac Surg. (2013) 44:454–61. 10.1093/ejcts/ezt062 23423918

[30] Donofrio MT Bremer YA Schieken RM Gennings C Morton LD Eidem BW. Autoregulation of cerebral blood flow in fetuses with congenital heart disease: the brain sparing effect. Pediatr Cardiol. (2003) 24:436–43. 10.1007/s00246-002-0404-0 14627309

[31] Limperopoulos C Tworetzky W McElhinney DB Newburger JW Brown DW Robertson RL. Brain volume and metabolism in fetuses with congenital heart disease: evaluation with quantitative magnetic resonance imaging and spectroscopy. Circulation. (2010) 121:26–33. 10.1161/CIRCULATIONAHA.109.865568 20026783 PMC2819908

[32] Clouchoux C du Plessis AJ Bouyssi-Kobar M Tworetzky W McElhinney DB Brown DW. Delayed cortical development in fetuses with complex congenital heart disease. Cereb Cortex. (2013) 23:2932–43. 10.1093/cercor/bhs281 22977063

[33] Miller SP McQuillen PS Hamrick S Xu D Glidden DV Charlton N. Abnormal brain development in newborns with congenital heart disease. N Engl J Med. (2007) 357:1928–38. 10.1056/NEJMoa067393 17989385

[34] Licht DJ Shera DM Clancy RR Wernovsky G Montenegro LM Nicolson SC. Brain maturation is delayed in infants with complex congenital heart defects. J Thorac Cardiovasc Surg. (2009) 137:529–37. 10.1016/j.jtcvs.2008.10.025 19258059 PMC2701902

[35] Dimitropoulos A McQuillen PS Sethi V Moosa A Chau V Xu D. Brain injury and development in newborns with critical congenital heart disease. Neurology. (2013) 81:241–8. 10.1212/WNL.0b013e31829bfdcf 23771484 PMC3770166

[36] Asschenfeldt B Evald L Yun HJ Heiberg J Østergaard L Grant PE. Abnormal left-hemispheric sulcal patterns in adults with simple congenital heart defects repaired in childhood. J Am Heart Assoc. (2021) 10:e018580. 10.1161/JAHA.120.018580 33745293 PMC8174332

[37] Sun L Macgowan CK Sled JG Yoo S-J Manlhiot C Porayette P. Reduced fetal cerebral oxygen consumption is associated with smaller brain size in fetuses with congenital heart disease. Circulation. (2015) 131:1313–23. 10.1161/CIRCULATIONAHA.114.013051 25762062 PMC4398654

[38] Ji W Ferdman D Copel J Scheinost D Shabanova V Brueckner M. *De novo* damaging variants associated with congenital heart diseases contribute to the connectome. Sci Rep. (2020) 10:7046. 10.1038/s41598-020-63928-2 32341405 PMC7184603

[39] Møller Nielsen AK Nyboe C Lund Ovesen AS Udholm S Larsen MM Hjortdal VE. Mutation burden in patients with small unrepaired atrial septal defects. Int J Cardiol Congenit Hear Dis. (2021) 4:100164. 10.1016/j.ijcchd.2021.100164

[40] Nolan EE Gadow KD Sprafkin J. Teacher reports of DSM-IV ADHD, ODD, and CD symptoms in schoolchildren. J Am Acad Child Adolesc Psychiatry. (2001) 40:241–9. 10.1097/00004583-200102000-00020 11211374

[41] Canino G Shrout PE Rubio-Stipec M Bird HR Bravo M Ramírez R. The DSM-IV rates of child and adolescent disorders in Puerto Rico: prevalence, correlates, service use, and the effects of impairment. Arch Gen Psychiatry. (2004) 61:85–93. 10.1001/archpsyc.61.1.85 14706947

[42] Döpfner M Breuer D Wille N Erhart M Ravens-Sieberer U. How often do children meet ICD-10/DSM-IV criteria of attention deficit-/hyperactivity disorder and hyperkinetic disorder? Parent-based prevalence rates in a national sample–results of the BELLA study. Eur Child Adolesc Psychiatry. (2008) (Suppl. 1):59–70. 10.1007/s00787-008-1007-y 19132305

[43] Pineda D Ardila A Rosselli M Arias BE Henao GC Gomez LF. Prevalence of attention-deficit/hyperactivity disorder symptoms in 4- to 17-year-old children in the general population. J Abnorm Child Psychol. (1999) 27:455–62. 10.1023/A:1021932009936 10821627

[44] Szomlaiski N Dyrborg J Rasmussen H Schumann T Koch SV Bilenberg N. Validity and clinical feasibility of the ADHD rating scale (ADHD-RS) a danish nationwide multicenter study. Acta Paediatr. (2009) 98:397–402. 10.1111/j.1651-2227.2008.01025.x 18775056

[45] Bilenberg N. The Child Behavior Checklist (CBCL) and related material: standardization and validation in Danish population based and clinically based samples. Acta Psychiatr Scand Suppl. (1999) 398:2–52. 10.1111/j.1600-0447.1999.tb10703.x 10687023

[46] Rescorla L Achenbach T Ivanova MY Dumenci L Almqvist F Bilenberg N. Behavioral and emotional problems reported by parents of children ages 6 to 16 in 31 societies. J Emot Behav Disord. (2007) 15:130–42. 10.1177/10634266070150030101

[47] Christiansen H Kis B Hirsch O Philipsen A Henneck M Panczuk A. German validation of the Conners Adult ADHD Rating Scales-self-report (CAARS-S) I: factor structure and normative data. Eur Psychiatry. (2011) 26:100–7. 10.1016/j.eurpsy.2009.12.024 20619613

